# Robot Evolutionary Localization Based on Attentive Visual Short-Term Memory

**DOI:** 10.3390/s130101268

**Published:** 2013-01-21

**Authors:** Julio Vega, Eduardo Perdices, José M. Cañas

**Affiliations:** Grupo de Robótica, Universidad Rey Juan Carlos, c/Camino del Molino s/n, 28943 Fuenlabrada, Spain; E-Mails: eperdices@gsyc.es (E.P.); jmplaza@gsyc.es (J.M.C.)

**Keywords:** visual attention, object tracking, active vision, visual localization

## Abstract

Cameras are one of the most relevant sensors in autonomous robots. However, two of their challenges are to extract useful information from captured images, and to manage the small field of view of regular cameras. This paper proposes implementing a dynamic visual memory to store the information gathered from a moving camera on board a robot, followed by an attention system to choose where to look with this mobile camera, and a visual localization algorithm that incorporates this visual memory. The visual memory is a collection of relevant task-oriented objects and 3D segments, and its scope is wider than the current camera field of view. The attention module takes into account the need to reobserve objects in the visual memory and the need to explore new areas. The visual memory is useful also in localization tasks, as it provides more information about robot surroundings than the current instantaneous image. This visual system is intended as underlying technology for service robot applications in real people's homes. Several experiments have been carried out, both with simulated and real Pioneer and Nao robots, to validate the system and each of its components in office scenarios.

## 1. Introduction

Computer vision research is growing rapidly, both in robotics and in many other applications, from surveillance systems for security to the automatic acquisition of 3D models for Virtual Reality displays. The number of commercial applications is increasing, such as traffic monitoring, parking entrance control, augmented reality video games, and face recognition. In addition, computer vision is one of the most successful sensing modalities used in mobile robotics. Cameras have been incorporated in the last years with robots as common sensory equipment. Vision has been used in robotics for navigation, object recognition, 3D mapping, visual attention, robot localization, *etc.* They are very cheap sensors and may provide much information to robots about their environment. However, extracting relevant information from the image flow is not easy.

Robots usually navigate autonomously in dynamic environments, and so they need to detect and avoid obstacles. There are several sensors which can detect obstacles in a robot's path, such as infrared sensors, laser range finders, ultrasound sensors, *etc.* When using cameras, obstacles can be detected through 3D reconstruction. Recovering 3D information has been the main focus of the computer vision community for decades. Stereovision methods are the classic ones, based on finding pixel correspondences between the two cameras and triangulation, despite their failure with untextured surfaces. Vision depth sensors like Kinect now offer a different technology for visual 3D reconstruction. In addition, structure from motion techniques build three-dimensional structure of objects by analyzing local motion signals over time, even from only one camera [1].

Moreover, many works have also been presented in vision-based navigation and control that generate robot behavior without explicit 3D reconstruction. The temporal occlusions of relevant stimuli inside the images are one hindrance to this approach. The control algorithm should be robust enough to face the lack of time persistence of relevant stimuli in images. This also poses a challenge when the objects lie beyond the current field of view of the camera. To solve this problem, some systems use omnidirectional vision. Others, like humanoids or robots with pan-tilt units, use mobile regular cameras that can be orientated at will and manage a visual memory of robot surroundings which integrates the information from the images taken from different locations. The visual representation of interesting objects around the robot beyond the current field of view may improve the quality of the robot's behavior as it handles more information when making decisions. This is where the problem of selecting where to look every time, known as gaze control, or *overt attention* [2,3] arises. Usually, the need to quickly explore new areas and the need to reobserve known objects to update their positions, and so on, influence this selection. This kind of attention is also present in humans: we are able to concentrate on particular regions of interest in a scene by movements of the eyes and the head, just by shifting attention to different parts. By focusing specifically on small regions which are important for the task at hand, and devoting as much effort as possible only to the relevant part, we avoid wasting processing power by trying to fully understand the whole surroundings.

Another relevant piece of information that can be extracted from images is robot location. Robots need to know their location inside the environment in order to unfold the expected behavior. Using robot sensors (especially vision) and a map, the robot may estimate its own position and orientation inside a known environment. Robot self-localization has proven to be complex, especially in dynamic environments and in those with much symmetry, where sensor values can be similar at different positions.

In this paper, we propose a visual perceptive system for an autonomous robot composed of three modules. First, a short-term dynamic visual memory of robot surroundings. It gets images from a mobile camera, extracts edge information, and offers a wider field of view and more robustness to occlusions than instantaneous images. This memory stores 3D segments representing the robot surroundings and objects. The memory contents are updated in a continuous coupling with the current image flow. Second, a gaze control algorithm has been developed to select where the camera should look every time. It manages the movement of the camera to periodically reobserve objects already stored in the visual memory, to explore the scene, and to test tentative object positions in a time-sharing fashion. These two modules working in conjunction build and update an attentive visual memory of the objects around the robot. Third, a visual localization algorithm has been developed which uses the current image or the contents of the memory to continuously estimate the robot position. It provides a robust localization estimation and has been specifically designed to handle symmetries in the environment.

The remainder of this paper is organized as follows. The second section reviews some of the related works. The third section presents the design of the proposed visual system, its components and connections. The following three sections describe its three building blocks: the visual memory component, visual attention algorithm, and localization component. The seventh section includes several experiments, both with simulated and real robots, performed to validate our system. Finally, some conclusions are made.

## 2. Related Works

Many issues have been tackled in the intersection of computer vision and robotic fields: vision-based control or navigation, vision-based map building and 3D representation, vision-based localization, object recognition, attention and gaze control, among others. We will review here some examples in the topics most related to the proposed visual perceptive system.

Regarding vision-based control and navigation, Remazeilles *et al.* [4] presented the design of a control law for vision-based robot navigation. The peculiarity of this control law is that it does not require any reconstruction of the environment, and it does not force the robot to converge towards each intermediary position in its path.

Recently, Srinivasan [5] presented a new system to increase accuracy in the optical flow estimation for insect-based flying control systems. A special mirror surface is mounted in front of the camera, which is pointing ahead instead of pointing to the ground. The mirror surface decreases the speed of motion and eliminates the distortion caused by the perspective. In this image, objects move slower than in the camera downwards, simplifying the optical flow calculation and increasing its accuracy. Consequently, the system increases the speed range and the number of situations in which the aircraft can fly safely.

Regarding visual map building, representation of the environment and navigation, Badal [6] reported a system for extracting range information and performing obstacle detection and avoidance in outdoor environments based on the computation of disparity from the two images of a stereo pair of calibrated cameras. The system assumes that objects protrude high from a flat floor that stands out from the background. Every point above the ground is configured as a potential object and projected onto the ground plane in a local occupancy grid called Instantaneous Obstacle Map (IOM). The commands to steer the robot are generated according to the position of obstacles in the IOM.

Goldberg [7] introduced a stereo vision-based navigation algorithm for the rover planetary explorer MER, to explore and map locally hazardous terrains. The algorithm computes epipolar lines between the two stereo frames to check the presence of an object, computes the Laplacian of both images and correlates the filtered images to match pixels from the left image with their corresponding pixels in the right one. This work also includes a description of the navigation module GESTALT, which packages a set of routines able to compute actuation, direction, or steering commands from the sensor information.

Gartshore [8] developed a map-building framework and a feature position detector algorithm that processes images online from a single camera. The system does not use any matching. Instead, it computes probabilities of finding objects at every location. The algorithm starts detecting the object boundaries for the current frame using the Harris edge and corner detectors. Detected features are backprojected from the 2D image plane considering all the potential locations at any depth. The positioning module of the system computes the position of the robot using odometry data combined with image feature extraction. Color or gradient from edges and features from past images help to increase the confidence of the object presence in a certain location. Experimental results in indoor environments set the size of the grid cells to 0.25 m × 0.25 m and the robot moved 0.1 m between consecutive images.

As we already mentioned, in autonomous robots it is important to perform a visual attention control. The cameras of the robots provide a large flow of data and the robot needs to select what is interesting and ignore what is not. There are two approaches to visual attention: *covert* and *overt* attention. The first one selects interesting areas inside the image for further processing [2,9–11]. The second one selects interesting areas in the environment surrounding the robot, even beyond the current field of view, and looks at them [12].

One of the concepts widely accepted in this area is the *salience map*. It is found in [2], as a covert visual attention mechanism, independent of the particular task to be performed. This bottom-up attention builds in each iteration the conspicuity map for each of the visual features that attract attention (color, movement, or edge orientations). There are competition dynamics inside each map and they are merged into a single representative salience map that drives the focus of attention to the area with highest value.

Hulse [13] presented an active robotic vision system based on the biological phenomenon of inhibition of return, used to modulate the action selection process for saccadic camera movements. They argued that visual information has to be subsequently processed by a number of cortical and sub-cortical structures that: (1) place it in context of attentional bias within egocentric salience maps; (2) implement the aforementioned inhibition of return (IOR) inputs from other modalities; (3) override voluntary saccades; and (4) influence basal ganglia action selection. Thus, there is a biological and sophisticated context-specific method for facilitating the most appropriate saccade as a form of attention selection.

The use of a camera in motion to facilitate object recognition was proposed by [14], and has been used, for example, to distinguish between different forms in the images [11]. Arbel and Ferrie presented in [15] a gaze-planning strategy that moves the camera to another viewpoint around an object in order to recognize it. Recognition itself is based on the optical flow signatures that result from the camera motion. The new measurements, accumulated over time, are used in a one-step-ahead Bayesian approach that resolves the object recognition ambiguity, while it navigates with an entropy map.

Grid-based probabilistic localization algorithms were successfully applied with laser or sonar data in small known environments [16]. They use discretized probability distributions and update them from sensor data and movement orders, accumulating information over time and providing a robust position estimation. Particle filters use sampled probability functions and extend the techniques to larger environments, using them even with visual data as input [17]. At the beginning, the maps were provided in advance, but as of late, the SLAM techniques tackle localization simultaneously with the map building. There are many particle filter based SLAM techniques. In addition, one of the most successful approaches is Mono-SLAM from Andrew Davison [18,19], which uses a fast Extended Kalman Filter for continuous estimation of 3D points and of camera position from relevant points in the images.

Genetic algorithms (GA) have been also proposed for robot localization tasks. For instance, in [20] the localization of a legged robot is modeled as the optimization of an objective function, and GA are used for that optimization using the vision data as input. In [21], Duckett combines an Extended Kalman Filter and a GA to locate a robot inside a known environment using ultrasonic sensors; this technique uses the EKF to obtain a seed, and then searches inside the seed neighborhood to find the most accurate solution with a GA. SLAM approaches have also included genetic algorithms, such as [22], where the best trajectory of a robot is calculated with a GA according to robot odometry and laser measurements.

In [23], Mariottini and Roumeliotis presented a strategy for active vision-based localization and navigation of a mobile robot with a visual memory where previously visited areas are represented as a large collection of images. It clarifies the location taking into account the sequence of distinctive images, while concurrently navigating towards the target image.

In [24], Jensfelt and Kristensen presented an active global localization strategy that uses Kalman filtering (KF) to track multiple robot pose hypotheses. Their approach can be used even with incomplete maps and the computational complexity is independent on the size of the environment.

## 3. Design

**Figure 1.**
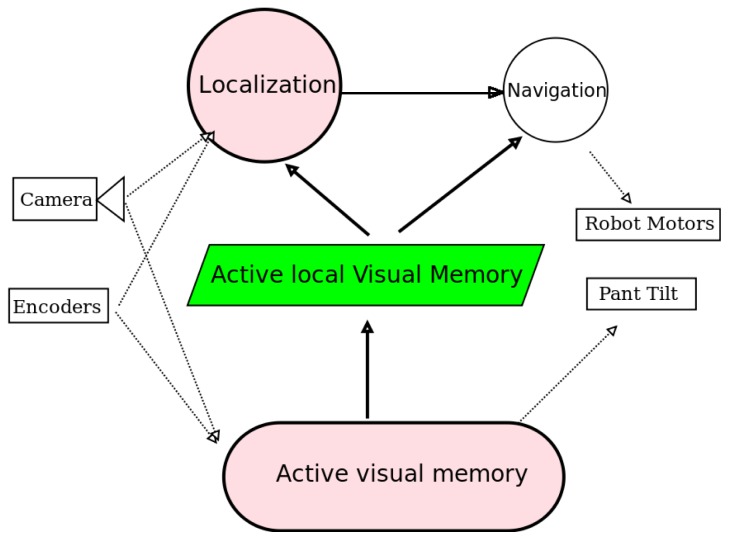
Block diagram of the proposed visual system.

The proposed perceptive system is designed for autonomous robots that use a mobile camera, like that on the head of humanoids or in robots with pan-tilt units. The block diagram of the robot control architecture is shown in Figure 1. The three building blocks (visual memory, gaze control and localization algorithm) have been grouped into two main software components: active_visual_memory and localization. They receive data from robot sensors, like camera and encoders, and extract refined information like description of the objects around the robot or the robot position. This information is provided to other actuation components like the navigation algorithm or other control units.

First, the active_visual_memory component builds a local visual memory of objects in the robot's surroundings. The memory is built analyzing each camera image looking for relevant objects (like segments, parallelograms, arrows, *etc.*) and updating the object features already stored in the memory, like their 3D position. The memory is dynamic and is continuously coupled with camera images. The new frames confirm or correct the object features stored in memory, like their 3D relative position to the robot, length, *etc.* New objects are introduced in memory when they appear in images and do not match any known object.

This memory has a broader scope than the camera field of view and objects in memory have more persistence than the current image. Regular cameras typically have 60 degrees of scope. This would be good enough for visual control but a broader scope may improve robot responses in tasks like navigation, where the presence of obstacles in the robot's surroundings should be taken into account even if they lie outside the current field of view.

This memory is intended as local and short-term. Relative object positions are estimated using robot's odometry. Being only short term and continuously correcting with new image data there is no much time to accumulate error in the object estimated relative position. Currently, the system only deals with objects on the floor plane and uses a single camera. It can be extended to any 3D object position and two cameras.

Second, in order to keep this short-term visual memory consistent with reality, the system has mechanisms to properly refresh and update it. The camera is assumed to be mobile, typically mounted over a pan-tilt unit. Its orientation may be controlled and changed during robot behavior at will, and so, the camera may look towards different locations even if the robot remains static. In order to feed the visual memory, an overt attention algorithm has been designed to continuously guide camera movements, choosing where to look every time. It has been inserted inside the active_visual_memory component and associates two dynamic values to each object in memory: *salience* and *life* (quality). Objects with low life are discarded, and objects with high salience are good candidates to look at.

The position of objects already in memory are themselves the foci of attention in order to refresh their perceived features. Random locations are also considered to let the robot explore new areas of its surroundings. In addition, new foci of attention may be introduced to check the presence of some hypothesized objects. For instance, once the robot has seen three vertices of a parallelogram, the position of the fourth one is computed from the visual memory and ordered as a tentative focus of attention for the camera.

Third, a vision-based localization algorithm has been developed in the localization component. It uses a population of particles and an evolutionary algorithm to manage them and find the robot position. The health of each particle is computed based on the current image or based on the current contents of the visual memory. The local visual memory provides information about the robot's surroundings, typically more than the current instantaneous sensor readings. In this way, the visual memory may be used as a virtual sensor and its information may be used as observations for the localization algorithm. Because of its broader scope, it may help to improve localization, especially in environments with symmetries and places that look similar according to sensor readings.

These two software components and the three building blocks will be described in detail in the following sections.

## 4. Local Visual Memory

The goal of our visual memory is to do a visual tracking of the various basic objects in the scene surrounding the robot. It must detect new objects, track them, update their relative positions to the robot and remove them from the memory once they have disappeared.

**Figure 2.**
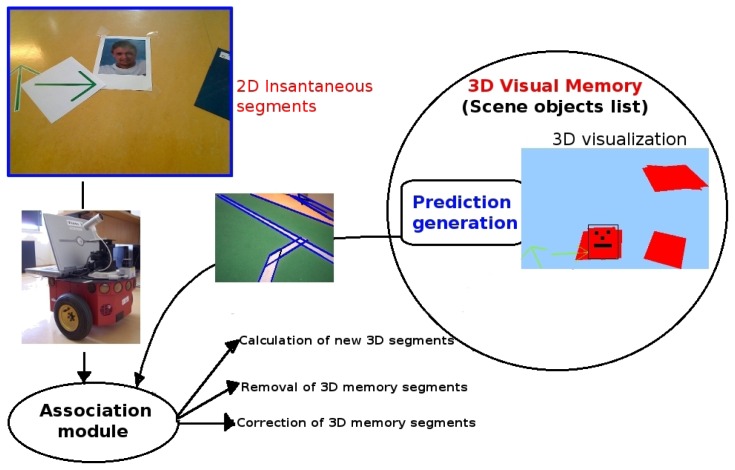
Modules of the Visual Memory.

Figure 2 shows the main modules of the visual memory building block. The first stage of the visual memory is a 2D analysis, which detects 2D segments present in the current image. These 2D segments are compared with those predicted from the current visual memory 3D contents. The 3D object reconstruction module places relevant 2D segments in 3D space according to the *ground-hypothesis* (we assume that all objects are flat on the floor as a simplifying hypothesis). Finally, the 3D memory module stores their position in the 3D space, updates or merges them with existing 3D segments, calculates perceptual hypotheses, and generates new predictions of these objects in the current image perceived by the robot.

The visual memory also creates a perceptual hypothesis with the stored items, allowing the system to abstract complex objects. For instance, it groups a set of 3D segments into the parallelogram concept if some geometric properties are held.

The visual memory has been coded inside the active_visual_memory software component, running iteratively at a frequency of 5 *Hz*. In each iteration, a motion prediction is made for objects in the visual memory according to robot encoders, images are acquired and displayed, image processing occurs and the memory contents are updated. To save computing power, robot odometry can be used to trigger the image processing and the system only analyzes frames when the robot has moved away a certain distance or angle from the position where the last image processing was done.

### 4.1. 2D Image Processing

**Figure 3.**
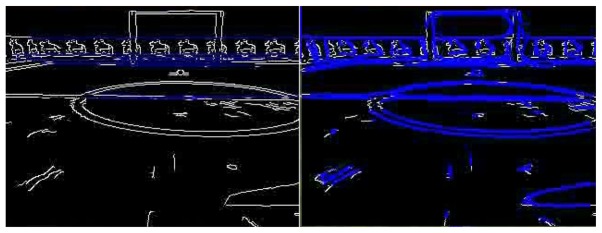
Differences between Canny+Hough (left) and Solis algorithm (right).

The main goal of this module is to extract 2D straight segments as a basic primitive to get object shapes. In prior implementations, we used the classic Canny edge filter and Hough transform to extract lines, but it was not accurate and robust enough. Usually its outcome was not fully effective: line segments were often disconnected as can be seen in Figure 3. In the latest releases, we replaced this with a Laplace edge detection and the Solis algorithm [25] for 2D extraction, improving the results. Solis's algorithm uses a compilation of different image processing steps such as normalization, Gaussian smoothing, thresholding, and Laplace edge detection to extract edge contours from input images. This solution is surprisingly more accurate, robust, faster and with less parameters for detecting line segments at any orientation and location than the widely used Hough Transform algorithm.

To implement these techniques, we use the OpenCV library. A comparison can be seen in Figure 3, where the Solis algorithm extracts many more 2D segments. The detected segments are shown as blue lines. While the Hough approach is able to recognize even a really small set of segments, the Solis one gets most of them. The floor used in this image is a textured surface and so some false positives appear.

**Figure 4.**
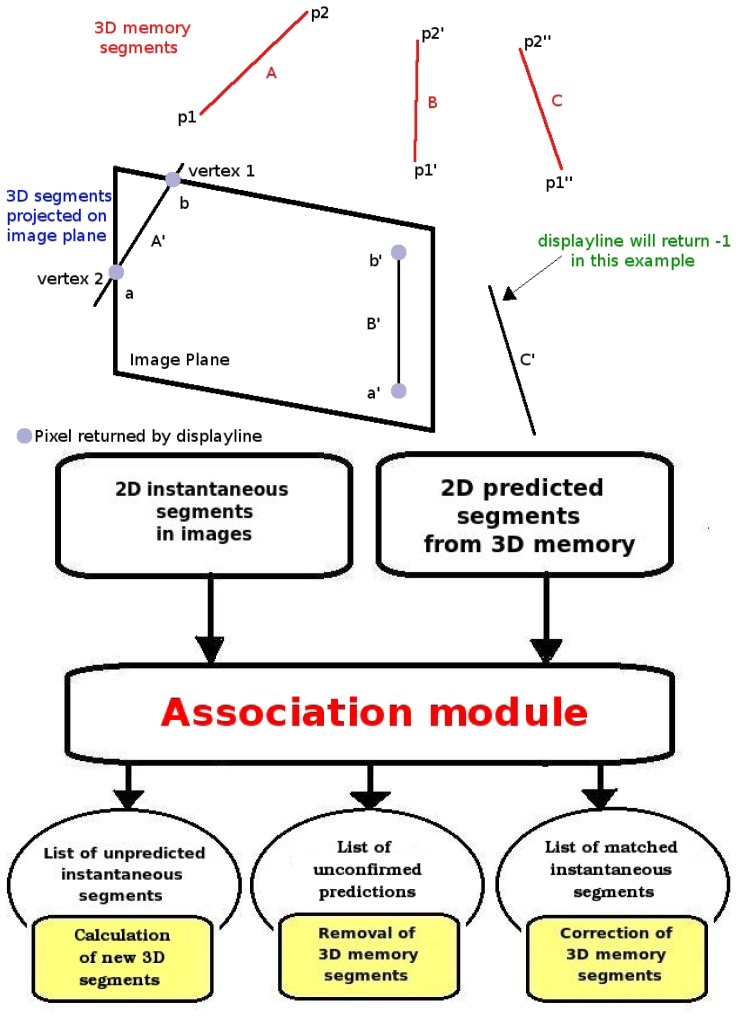
3D projection on the image plane (left) and matching between predicted and observed segments (right).

The 2D analysis system is connected directly to the 3D visual memory contents to alleviate the computational cost of image analysis. Before extracting features of the current image, the system predicts inside the 2D image the appearance of those objects already stored in the 3D memory which are visible from the current position. We use our *projective geometry* library to do this. Each stored 3D visible object is projected on the image plane as shown in Figure 4 (left). The system refutes/corroborates such predicted segments, comparing them with those coming from the 2D analysis on observed images. This comparison provides three sets of segments, as seen in Figure 4: those that match with observations, those that do not match, and observed segments that are unpredicted, that is, without an homology in the 3D memory. Matched segments will be used to update the information of their homology in 3D memory. Unpredicted observed segments will be located in 3D and inserted in the visual memory as new 3D segments.

### 4.2. Reconstruction with 3D Segments

This module is responsible for obtaining 3D instantaneous information from 2D segments and objects in the current image. To do this, we rely on the idea of *ground hypothesis*, assuming that all the objects are flat on the floor. This simplifying assumption makes it easier to estimate the third dimension from a single camera. This module can be replaced with other 3D techniques and full 3D estimation in case of a stereo pair.

There are four relevant 3D coordinate systems in this approach. First, there is the absolute coordinate system; its origin lies somewhere in the world where the robot is moving. Second, there is the system located at the base of the robot. The robot odometry gives its position and orientation with respect to the absolute system, with some noise. Third, there is the system relative to the base of the pan-tilt unit to which the camera is attached. It has its own encoders for its position inside the robot at any given time, with pan and tilt movements with respect to the base of the robot. Fourth, there is the camera relative coordinate system, displaced and oriented in a particular mechanical axis from the pan-tilt unit.

The visual memory is intended to be local, and it contains accurate relative position of objects around the robot, despite their global world coordinates being wrong. For visual memory purposes, the robot position is taken from the encoders, so it accumulates error as the robot moves along and deviates from the real robot location in the absolute coordinate system. The segment and object positions computed from images take into account such robot location. This way, the segment and object positions in visual memory are absolute, accumulate error and deviate from their real coordinates in the world, but, subtracting the robot position measured from the encoders, their relative positions are pretty accurate. There is no problem with this as long as the visual memory does not intend to be a global map of the robot scenario. The visual memory is going to be used from the robot point of view, extracting from it relative coordinates of objects: for local navigation and as a virtual observation for the localization algorithms.

**Figure 5.**
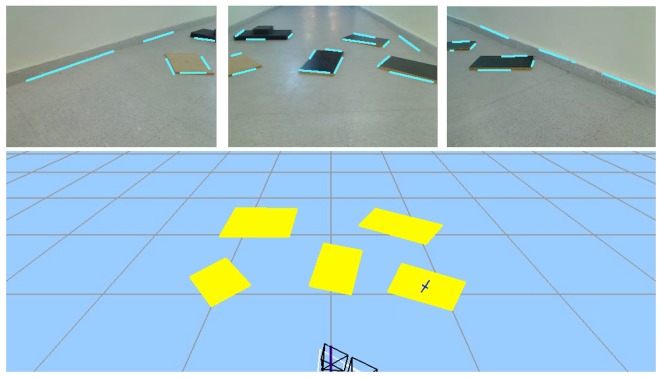
Scene situation with three instantaneous images and 3D scene reconstruction.

Once we have the 3D segments, and before including them on the 3D memory, some postprocessing is needed to avoid duplicates in memory due to noise in the images. This postprocessing compares the relative position between segments, as well as its orientation and proximity, maybe merging some of them. The output is a set of observed 3D segments situated on the robot coordinate system. Figure 5 shows the segments detected in the current image, the predicted segments from the current position, and all the objects (parallelograms) formed by these segments and recognized by the system in the 3D observed scene.

### 4.3. Inserting Segments into the 3D Visual Memory

3D visual memory comprises a dynamic set of lists which stores information about the different types of elements present in the scene (position, type or color). The most basic element is the 3D segment. The visual memory also can establish relationships between them to make up more complex elements such as arrows, parallelograms, triangles, circles or other objects.

As mentioned before, the 2D analysis returns different subsets of segments, as the result of comparison between observed and predicted segments from 3D memory. If a segment is identified in the current image and it does not match the predictions, the system creates a new one in 3D. For matched segments they are located in 3D merged with the 3D segments already stored in the visual memory. They will be nearby segments with similar orientation and so the system combines these segments into a new one taking the longest length of its predecessors, and the orientation of the more recent, as probably it is more consistent with reality (the older ones tend to have more noise due to errors in robot odometry). The 3D segments have an attribute named *uncertainty* which increases while the segment remains in memory and is also taken into account and updated. To make this fusion process computationally lighter, the system has a 3D segment cache of the full 3D segment collection with only the segments close to the robot (in a radius of 4 m).

As will be described later, the 3D segments have an attribute named *life* which decreases while the segment remains in memory and is not matched with any observation. Every time there is a matching, the *life* of the corresponding 3D segment is increased. If the uncertainty on a 3D segment falls below a given threshold, it is deleted from visual memory.

### 4.4. Complex Primitives in Visual Memory

Visual memory manages simple 3D segments and other primitives like parallelograms. The segments and their corresponding vertices are used to detect parallelograms checking the connection between them and the parallelism conditions. The analysis of the angles formed by each segment provides information about how the segments are connected to each other. The visual memory can estimate the position of a possible fourth vertex using the information about edges and the other three vertices. In addition, the parallelogram primitive can be used to merge incomplete or intermittent segments. This capability makes our algorithm robust against occlusions, which occur frequently in the real world.

**Figure 6.**
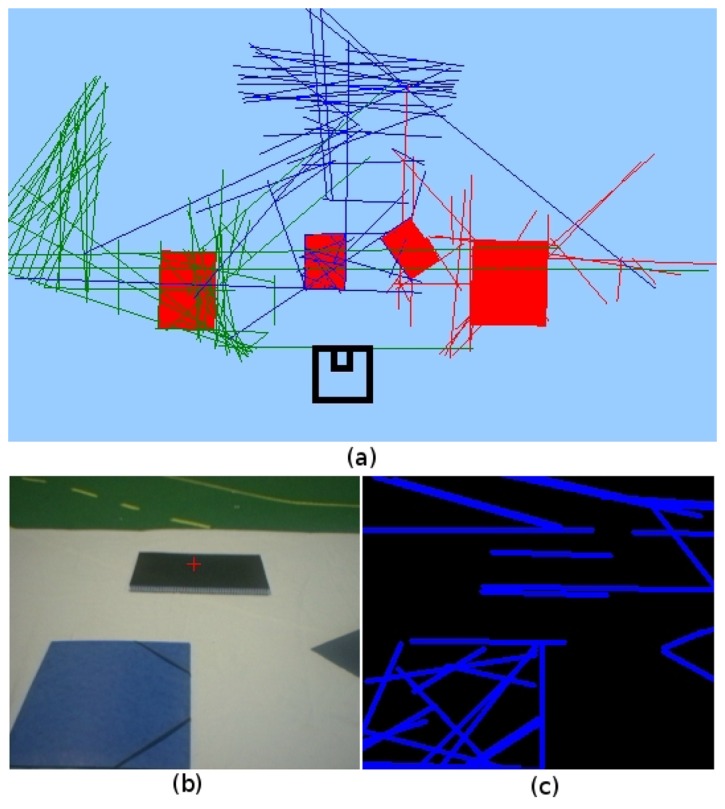
Complex primitives in visual memory: parallelograms with occlusion.

Figure 6 illustrates an example of occlusion that is successfully solved by our algorithm. The Figure 6(b) image shows the observation of incomplete parallelograms. The results of reconstruction of parallelograms can be seen in Figure 6(a), with the extracted four parallelograms spread on the floor. The robot, after several snapshots with incomplete parallelograms, captures the real ones in 3D avoiding the noise in the observations. In this example, one threshold of the Solis detection algorithm has been increased to demonstrate the robustness of our algorithm against noise and incomplete detections of complex objects in the scene. From the input image in Figure 6(b), there are many noisy 2D instantaneous segments extracted (Figure 6(c)) and therefore in the corresponding 3D segments (Figure 6(a)), but parallelograms are perfectly detected in the visual memory (red objects in Figure 6(a)).

## 5. Visual Attention

The second building block of the proposed visual perception system is the visual attention. It uses two object attributes: *salience* and *life* to decide where to look at every moment and to forget objects when they disappear from the scene. In addition, this block includes a mechanism to control the camera movements for object tracking and exploring of new unknown areas from the scene.

### 5.1. Gaze Control: Salience Dynamics

Each object in the visual memory has its own 3D coordinates in the scene. It is desirable to control the movement of the pan-tilt unit towards that position periodically in order to reobserve that object. To do so, we introduced the dynamics of salience and attention points. The position of different 3D segments and objects in the visual memory are attention candidate points. Each one has a salience that grows over time and vanishes every time that that element is visited (providing so the Inhibition of Return), following the Equation (1):

(1) $$\text{Salience}\left(t\right)=\{\begin{array}{ll} 0 & \text{if object attended}\\ \text{Salience}\left(t-1\right)+1 & \text{otherwise} \end{array}$$

The system continuously computes the salience of all attention points and chooses the most salient one to control the gaze. The pan and tilt orders that make the camera look at it are then computed and commanded to the pan-tilt unit. When a point is visited, its salience is set to 0. A point that the system has not visited recently gets more salience than one which has just been attended. If the salience is low, it will not be visited now. The system is thus similar to the behavior of a human eye, as pointed by biology studies [9]: when the eye responds to a stimulus that appears in a position that has been previously treated, the reaction time is usually higher than when the stimulus appears in a new position.

After a while, the most salient point is recomputed and so the focus of attention is changed, implementing a kind of time-sharing gaze control. The designed algorithm allows for an alternation of the focus of the camera between the different objects in the scene according to their salience. We assume that an object will be found near the location where it was previously observed the last time. If the object motion were too fast then the reobservations would not match with the current object location.

In our system, all objects have the same slope in the saliency dynamics—the same preference of attention—and so all of them are observed with the same frequency. If we assigned different rates of growth of salience, we could have different priorities for the objects, causing the pan-tilt unit to look more often at the objects whose salience grows faster.

### 5.2. Tracking of a Focused Object

**Figure 7.**
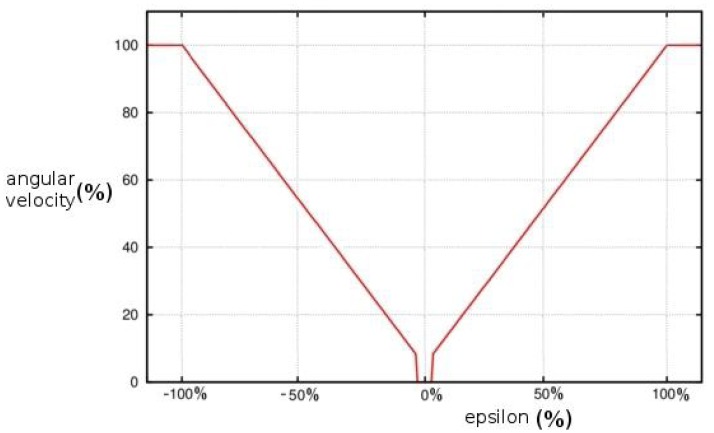
P-controller mechanism.

When the gaze control chooses the attention point of a given object, the system will look at it for a certain time (3 seconds), tracking it if it moves spatially. For this tracking, we use two proportional controllers to command the pan and tilt speeds and thus continually keep that object in the center of the image. As can be seen in Figure 7, the controller follows the Equations (2) and (3), where: *K_p_* is the P control gain, *T_t_* is the Tilt of the target, *T* is the current Tilt, *P_t_* is the Pan of the target, *P* is the current Pan, *M_t_* is the maximum Tilt acceptable error, *M_p_* is the maximum Pan acceptable error, ∊*_p_* is (*P_t_* − *P*) and ∊*_t_* is (*T_t_* − *T*).

(2) $$v\left(\text{Pan}\right)=\{\begin{array}{ccc} 0 & \text{if} & {∊}_{p}\;<0.3\\ K_{p}\cdot \left(P_{t}-P\right) & \text{if} & 0.3\;\leq \;{∊}_{p}\;<M_{p}\\ K_{p}\cdot M_{p} & \text{if} & M_{p}<\;{∊}_{p} \end{array}$$

(3) $$v\left(\text{Tilt}\right)=\{\begin{array}{ccc} 0 & \text{if} & {∊}_{t}\;<0.1\\ K_{p}\cdot \left(T_{t}-T\right) & \text{if} & 0.1\leq \;{∊}_{t}\;<M_{t}\\ K_{p}\cdot M_{t} & \text{if} & M_{t}<\;{∊}_{t} \end{array}$$

### 5.3. Exploring New Areas of Interest

The robot capability to look for new objects in the scene is interesting. This search is especially convenient at the beginning of operation, when there are many unknown areas of the scene around the robot with objects of interest. For that search our system periodically inserts (every *forcedSearchTime*) attention points with high salience in the visual memory. Due to its high salience, they will be quickly visited with the camera and so that location will be checked whether or not any object of interest is found around it. In such a case, that object will enter into the visual memory and into the regular gaze sharing.

The scanning points can be of two types: random and systematic ones. Random points are distributed uniformly within the pan-tilt range. Systematic scanning points follow a regular pattern to finally cover the whole scene around the robot. With them, the system ensures that eventually all areas of the scene will be visited.

There will be a proliferation of points of exploration in the beginning, when there are few objects in memory to reobserve. As the robot discovers objects, the desire to explore new areas will decrease in proportion to the number of already detected objects.

### 5.4. Representation of the Environment: Life Dynamics

As already mentioned in previous sections, the objects may eventually disappear from the scene, and then they should be removed from the memory in order to maintain coherence between the representation of the scene and the reality. To forget such old elements, we have implemented the *life* dynamics that follows the Equation (4).

(4) $$\text{Life}\left(t\right)=\{\begin{array}{c} \min\left({\mathrm{MAX}}_{\text{LIFE}},\text{Life}\left(t-1\right)+\Delta \right)\\ \text{if object observed}\\ \text{Life}\left(t-1\right)-1\\ \text{otherwise} \end{array}$$

*Life* of unobserved 3D segments or objects decreases over time. Every time an object or 3D segment is observed in the images (just because the gaze control visits it or visits one nearby object), its *life* increases, with a maximum saturation limit. This way when the *life* of an object exceeds a certain threshold, that means it is still on the scene, whereas when it is below it, that means it has left and so it is deleted from visual memory.

### 5.5. Attention Module Operation

The proposed visual attention module is fully *bottom-up*. The objects surrounding the robot guide the movements of the camera, just to reobserve them, to track them or to explore the environment looking for them. Periodically, the system updates the salience and life attributes of the objects that are already stored in memory following previous equations. It checks whether any of them is already outdated, because its life is below a certain threshold. If not, it increases its salience and reduces its life.

It has been implemented following a *finite-state machine* that determines when to execute the different steps of the algorithm: select next goal (state 0), complete the saccadic movement (state 1), analyze image (state 2) and track the object (state 3). In the initial state, the system asks whether there is any attention point to look at (in case we have an object previously stored in memory) or not. If so, it goes to state 1. If not, it inserts a new scanning attention point into memory and goes back to state 0. In state 1, the task is to complete the movement towards the target position. Once there, the automata goes to stage 2, and it analyzes whether there are relevant objects in the images or not. After a while, it returns to state 0 and starts again.

## 6. Evolutionary Visual Localization

We have designed a new approach to solve robot self-localization specifically designed to deal with symmetries. It is an evolutionary algorithm, a type of meta-heuristic optimization algorithm that is inspired by the biological evolution.

In this kind of algorithm, candidate solutions are so-called “individuals,” which belong to a population that evolves over time using genetic operators, such as mutation or crossover. Each individual is a tentative robot position (*X, Y, θ*) and it is evaluated with a quality function which calculates its “health,” that is, a measure to know how good its localization is with regard to the optimal solution. We have defined two different health functions, one based on instantaneous measurements of robot sensors and another one based on the visual memory contents.

Races are sets of individuals around a given location; they perform a fine-grain search around it. The algorithm has *R* races which compete among each other to be the race containing the best pose estimation. Each race has several associated parameters like the number of iterations without being deleted, the number of iterations containing the best pose estimation, *etc.*

**Figure 8.**
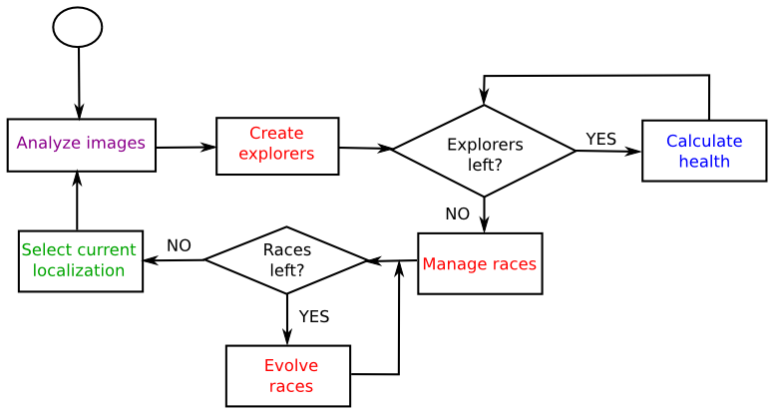
Basic diagram of evolutionary algorithm.

The main idea of the algorithm consists of keeping several races competing against each other in several likely positions. In case of symmetries from observations, the algorithm will create new races on various positions where the robot might be located. After some iterations, predictably, new observations will provide information to reject most of the races and the algorithm will obtain the real robot pose from the best race. On each iteration of the algorithm, it performs several steps to estimate the current robot pose (Figure 8). They are described in detail in the following sections.

- Health race calculation using the information obtained after analyzing images.
- Explorer creation: We spread randomly new individuals, explorers, with the aim to find new candidate positions where new races could be created.
- Race management: We create, merge or delete races depending on their current state.
- Race evolution: We evolve each race by using genetic operators. Besides, if the robot is moving, we update all races taking into account this movement.
- After calculating each race health, we will select one of them to set the current robot pose estimation.

We have created two branches of the proposed evolutive localization algorithm. The first one takes data directly from the current camera image. Its health function is described in Section 6.2. The second one takes data from the local visual memory, its 3D segments. Its health function is described in Section 6.3. Current implementation of the visual memory component only creates 3D segments lying on the floor, but the localization algorithm is ready for accepting 3D segments in any position and orientation.

### 6.1. Analyzing Images

**Figure 9.**
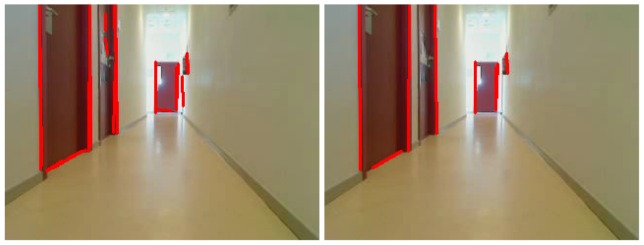
Image analysis before merging (left) and after merging (right).

When the localization algorithm uses the instantaneous camera images, it first detects lines inside the images, following the same technique described in Section 4.1. This time, the algorithm does not discard segments over the horizon, as it does not require that objects lie on the floor. Some postprocessing in the image is performed to clean and refine the segments. The postprocessing associates a label to each segment depending on the main colors on both sides of the segment. Segments with an unknown type are rejected. After labeling each segment, the algorithm tries to merge segments with the same type if their extremes are close to each other, joining consecutive segments together (Figure 9). Segments that are too small are discarded.

**Figure 10.**
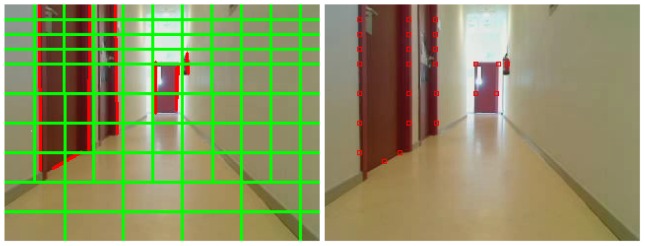
Image grid (left) and points selected (right).

Instead of using these lines directly as input data, the algorithm divides them into sampling points to make the comparison between lines easier, as it will be explained in Section 6.2. We created a grid with different cell sizes and we only save a new point where the lines detected intersect with this grid (Figure 10). The size of these grid cells changes because we want to analyze the upper part of the image more deeply than the lower one, since further objects will be at the top of the image and its resolution will be smaller. All these selected sampling points will be the input data to calculate the health of each individual.

### 6.2. Health Calculation from Instantaneous Images

The health of an individual placed at a certain location is computed comparing the theoretical set of visible objects and segments from that location (theoretical observation) with objects and segments currently observed (real observation). The more similar the predicted segments and the observed ones are, the more likely such a location is the correct one.

**Figure 11.**
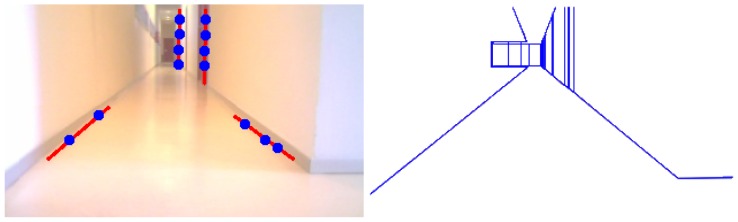
Detected lines in current image and theoretical image.

The theoretical observations are generated *ad hoc* for each particular location, projecting lines from the environment map into the camera placed at that location (Figure 11). It contains the lines the robot would see if it were placed at that location (Figure 11 (right)). It is assumed that the map of the environment is known.

For each sampling point in the observed lines (Figure 11 (left)) the Euclidean distance *d_i_* in pixels to the closest theoretical line with the same label is computed. After calculating *d_i_* for all points, the individual's health is computed as the average distance, following the Equation (5), where *N* is the number of points and *M* is the maximum distance allowed in pixels (set to 50 pixels for a 320 × 240 image size).

(5) $$H=1-\frac{\sum_{i=0}^{N}\frac{d_{i}}{N}}{M}$$

We will show in Section 7.2.4. several experiments to analyze the health function behavior in different situations.

### 6.3. Health Calculation with Visual Memory

In the of using the visual memory, we do not need to analyze each image, just the current visual memory contents from the active_visual_memory component, that is, the set of 3D segments inside, relative to our robot. Thus we cannot compare lines in image as we did before. Besides, we have to take into account that lines may not be detected completely or they may be divided into several small lines. Therefore, to calculate the health of an individual, we cover all lines belonging to the visual memory, for each line we get its extremes and calculate the Euclidean distances *d_j_s__* and *d_j_e__* to the closest theoretical line with the same label. This is similar to health function with instantaneous images but in 3D. After calculating *d_j_s__* and *d_j_e__* for each line, we can calculate the health as follows, where *N* is the number of lines and *M* is the maximum distance allowed in meters (set to 0.5 meters).

(6) $$H=1-\frac{\sum_{i=0}^{N}\frac{\left(\frac{d_{j_{s}}+d_{j_{e}}}{2}\right)}{N}}{M}$$

### 6.4. Explorer Creation

Explorers are individuals that do not belong to any race and that try to find likely positions with good health. There are two ways to spread explorers around the environment: randomly or following a predesigned pattern of search positions. In order to be truly general and avoid the overfitting of the algorithm, we chose the random approach.

When explorer creation is performed, the algorithm creates *E* explorer individuals, calculates their health and arranges them according to their health. Then, the best *M* explorers are promoted to become a candidate to create a new race. The number of explorers created changes dynamically depending on the current algorithm status: if the robot is lost, *E* is increased, and it decreases if its location is reliable. Moreover, since explorer creation is time consuming, when current location is reliable, the explorer creation is not executed at each algorithm iteration, but once every certain iteration.

### 6.5. Race Maanagement

In the long term, the algorithm manages several races and needs a policy to decide when to create a new race, delete it, or merge two races. The algorithm uses two parameters of each race for that: “victories” and “age.” A race increases its “victory” counter in an iteration when its health is higher than that of the rest of races. At the race creation, this parameter is set to 0. This number can also decrease if the winner in a given iteration is another race. This parameter is useful to select the race that finally sets the current robot pose estimation in each iteration. The age parameter shows the number of algorithm iterations since its creation. It has been created with two objectives. First, it preserves new races from dying too soon to avoid creating races that will be deleted in the next iteration. When a race is created this race cannot be deleted or replaced (although it may be merged) until its age reaches 3. Second, it avoids deleting a race because of wrong sensor information. If a race has had the highest health in an iteration, the algorithm will not delete it at least until 9 iterations after that. This provides some stability to races and avoids the continuous creation and deletion of races.

Our approach has a maximum number or races *R* that avoids the exponential increasing of computation time related to the number of races. Whenever the explorer creation is executed and there are new candidates to become a race, we have to decide when to create a new race and when to replace an existing one. If the maximum number of races *R* has not been reached, for each candidate, we find out if an existing race is located in the same position (*X, Y, θ*). In such a case, we do not try to create a new race, but we assume that the existing race already represents this candidate, and its age is increased. If candidates are innovative enough, we create a new race. If the maximum number of races is reached, the candidate will replace a victim race if the health of the candidate is greater than that of the victim and the victim's victories parameter falls below 0. If no race can be replaced, candidates are ignored.

In case two races evolving towards the same location, we consider that they have led to the same solution, so we merge them. This merging consists of deleting the race with lower victories. If both have the same victories, we keep the best race according to its health.

A race will be deleted when its victories are 0 and its health is below 0.6. In such a case, the race is no longer at the real robot pose; the algorithm considers it wrong and deletes it.

### 6.6. Race Evolution

When a race is created from an explorer, all its individuals are created applying a random thermal noise to the explorer who created the race. From then on, in the next iterations, its individuals evolve through three genetic operators: elitism, crossover and mutation. With elitism, the algorithm selects the best individuals of each race, arranging them according to their health. They are saved in the next iteration without any change. With crossover, the algorithm randomly selects several pairs of individuals and calculates their average with their values (*X, Y, θ*) for the next iteration. With mutation, the algorithm selects an individual randomly and applies a thermal noise to its position and orientation.

Furthermore, in case the robot has moved since the last iteration, we apply a motion operator to all races and individuals at the beginning of each iteration using robot odometry. Once all the individuals of each race have evolved, the algorithm calculates the final pose of the race as the average of its elitist individuals,= in order to avoid abrupt changes.

### 6.7. Selecting the Robot Pose Estimation

After evaluating all the existing races, the algorithm chooses one of them in each iteration to be the current pose of the robot. The selected race will be the one with more victories and its pose will determine the robot pose calculated by the algorithm.

The first step is selecting the race with greatest health in the current iteration (*R_i_*). If the race selected was the same in the previous iteration (*R_p_*), we increase the victories of *R_i_* and we decrease the victories of the rest. However, if *R_i_* and *R_p_* are different, we only change the races victories if the difference between *R_i_* health and *R_p_* health is greater enough. This distinction is made because we want race changing to be difficult if *R_p_* has been the selected race during a lot of iterations. With this behavior, we try to help the races which have been selected in the previous iterations and we only change the winner race when the health difference is big enough (which would mean that the current localization is wrong).

## 7. Experiments

To verify our different approaches of visual memory, visual attention and visual localization, we conducted several experiments. Our experimental real platforms were an ActivMedia Pioneer 2DX robot equipped with a Logitech Autofocus camera (2 megapixels) and a Nao Robot from Aldebaran Robotics (v3 model). In addition, we have used Gazebo 0.9 as robot simulator. All our experiments are implemented on C++ with Jderobot robotics software platform, which uses ICE as communication middleware.

Our visual system is primarily intended as underlying technology for service robot applications in the homes of real people. The service robot that uses vision will need to perceive obstacles around it, even beyond the current field of view, and to know its position in the house to launch proper actions. For the real tests, we used our attentive visual memory and localization algorithm in an office scenario with doors, corridors, lights, *etc.* without any robot-specific landmark. We have preferred this real life environment, as is, over simplified ones like those in the RoboCup. Moreover, the features of the RoboCup competition, with highly dynamic situations around the robot, are different from those in real homes. The proposed algorithms have been tested so far on a moving robot in static or low dynamism scenarios, with few moving objects around or slow movements.

### 7.1. Attentive Visual Memory Experiments

#### 7.1.1. Robot in the Middle of a Room

**Figure 12.**
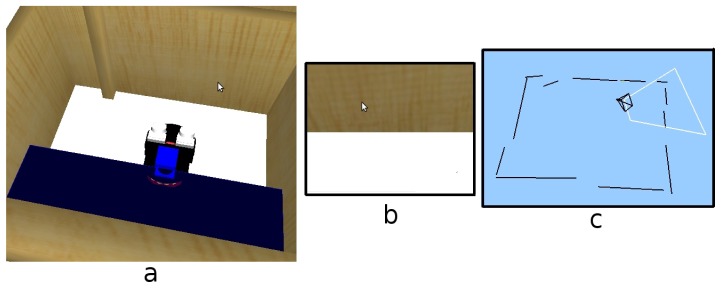
(**a**) Situation; (**b**) Instantaneous image; (**c**) Short-term memory.

For the first experiment, the robot is in the middle of a room (see Figure 12(a)). Then the robot turns around itself. Figure 12(b) shows an instantaneous view from the room, where the robot is able to detect a simple line on the floor. After a few seconds, the robot has turned a full circle, having stored all the information about its surrounding environment. Thus, we can see in Figure 12(c), how the short-term memory provides more information than an instantaneous image.

#### 7.1.2. Robot Navigating a Curve

**Figure 13.**
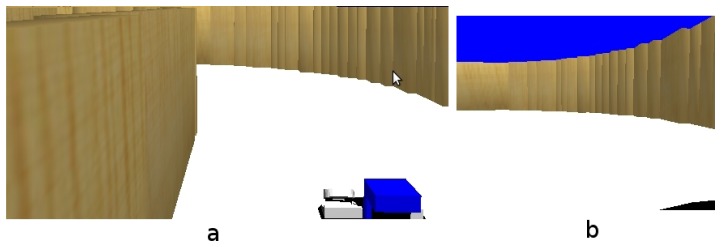
(**a**) Situation; (**b**) Current onboard image.

**Figure 14.**
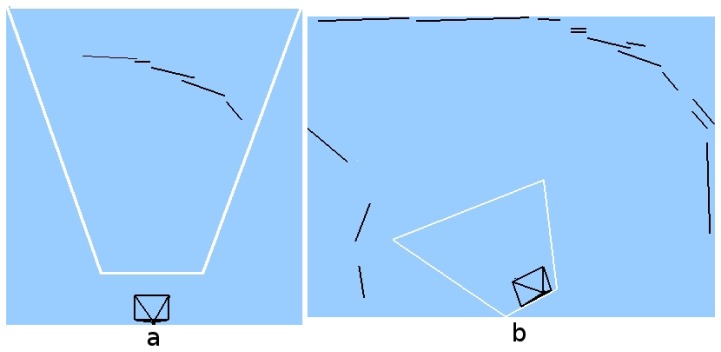
(**a**) Information in current field of view; (**b**) Short-term memory.

This experiment shows how the robot is unable to navigate using only the instantaneous information received from the camera. The situation is shown in Figure 13(a), the robot approaches to a curved area, while navigating through a corridor. If the robot used only instantaneous images (Figure 13(b), it would be able to see only just some lines in front of itself (Figure 14(a), but with short-term memory, it can observe that the path in front of itself is a curve (Figure 14(b). In addition, the robot can quickly explore its surrounding environment thanks to the visual attention mechanism, which forces the system to explore unknown areas of the environment.

#### 7.1.3. Robot Occlusions

Here, the situation is presented to solve a temporary occlusion. This happens very often in real environments where there are dynamic objects which can obstruct the robot's field of view.

**Figure 15.**
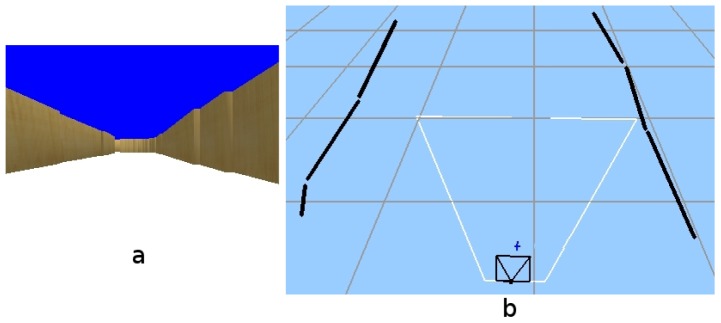
(**a**) Situation; (**b**) Short-term memory achieved after a while.

The initial situation is shown in Figure 15(a). After a few seconds, the robot has recovered environment information thanks to the short-term memory and the visual attention system (as displayed in Figure 15(b)).

**Figure 16.**
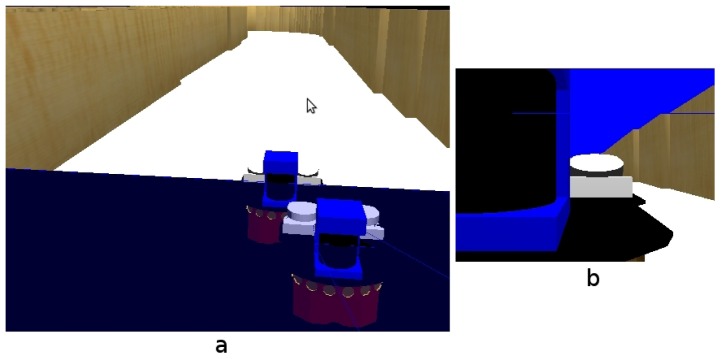
(**a**) Situation; (**b**) Field of view.

**Figure 17.**
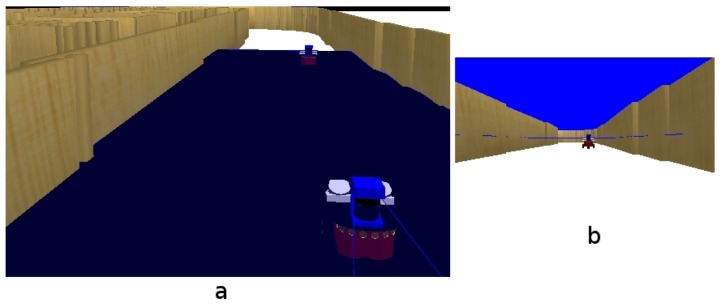
(**a**) Situation; (**b**) On board current image.

Then another robot appears, as shown in Figure 16(a), temporarily occluding the field of view of our robot (Figure 16(b)), so the robot is unable to see anything. This situation continues for some time until the second robot moves away from our robot (Figure 17(a,b)).

This situation is solved by our system with the persistence of the short-term memory, and so, the robot can make control decisions taking into account information of areas beyond the robot that is occluding its camera. As we presented before, the memory is continuously refreshed and updated over time. If it is inconsistent, that is, if what the robot sees does not match the information stored in its memory, the system has some persistence before changing the memory contents.

#### 7.1.4. Attentive Visual Memory on a Humanoid Robot

**Figure 18.**
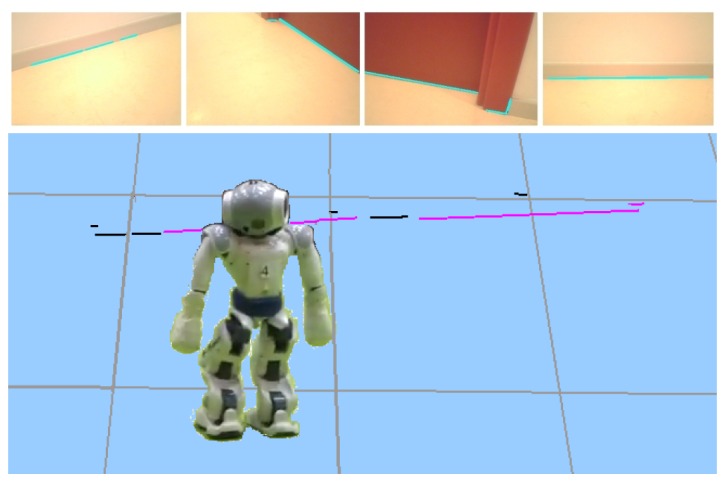
Visual memory with 3D segments coming from four images of robot surroundings.

In this experiment, we tested our whole system, including the visual memory, visual attention algorithm, and visual localization on a real robotic platform such as a Nao humanoid robot. The robot is in the middle of our department corridor gathering information about its environment. It is able to move autonomously around the corridor; furthermore, it is moving its neck in order to detect all segments in a few seconds. We can see in Figure 18 some snapshots and the short-term memory built with them.

The local visual memory updating and the attention module run in iterations. The average iteration time including all computations (2D image processing, matching with predictions, 3D reconstruction, segment insertion, complex primitives management, *etc.*) was 80 ms.

### 7.2. Visual Localization Experiments

We have performed several experiments to validate the evolutionary algorithm as well, especially with real robots. Our localization algorithm is map based. The map in these experiments has been introduced in the robot as a collection of 3D segments at certain positions, stored in a file. First of all, we need to explain why we have designed an algorithm to specifically deal with symmetries instead of deploying other widely used algorithms such as particle filters.

#### 7.2.1. Testing MCL Algorithm Behavior

**Figure 19.**
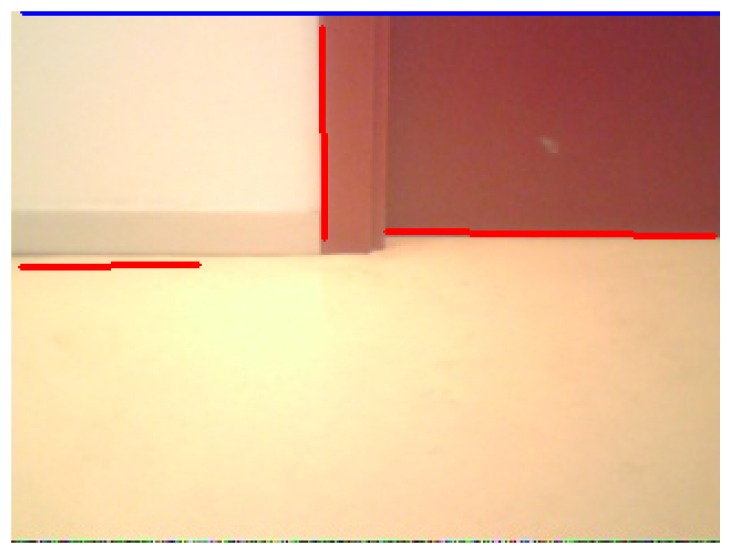
Observation taken to update MCL particles.

**Figure 20.**
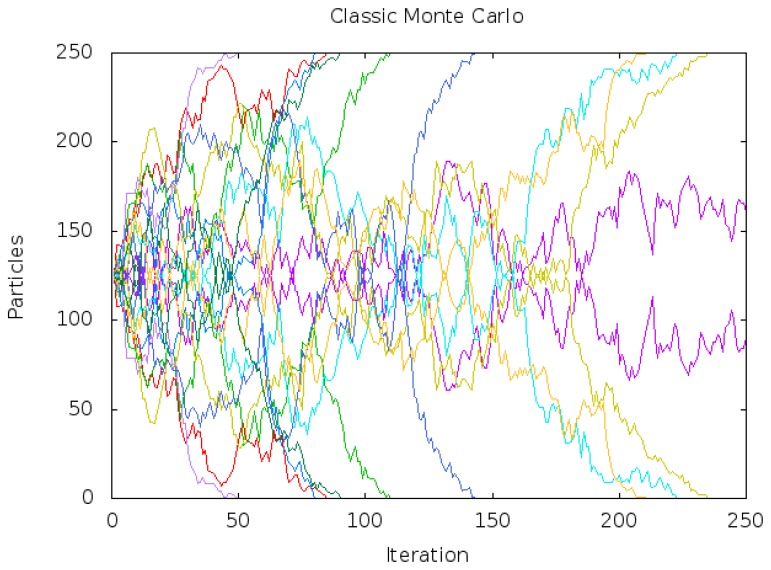
Monte Carlo particles evolution.

We have implemented a Monte Carlo localization algorithm [26] with the same health calculation (observation model) and motion operator (motion model) that we explained in Section 6. We performed a theoretical experiment to analyze MCL behavior within symmetric environments. We placed two particle populations with the same number of particles (125) in front of two identical doors of our environment, placing each populations at the same distance from each door and updating MCL particles with the same observation (Figure 19):

Since both populations are located at the same distance from each door, and observations are identical, MCL should keep both populations until new information is obtained. However, we show in Figure 20 the real behavior of the algorithm, where one of the populations is always ruled out after a while. In the vertical axis, the number of particles of each race is displayed (different colors for different runs). The horizontal axis displays the iteration number. In our experiments, MCL has been able to keep both populations with enough particles only once for more 250 iterations (about 30 seconds). In most of the runs, one of the two populations eventually gained all the particles of the algorithm.

This bias towards one of the populations happens because MCL picks up the particles of the next population randomly in the “roulette” step. By chance, this randomness may choose more particles from one population than from the other, and then this last population has even fewer probability of providing samples at the next iteration, decreasing gradually the particles coming from it, until such population is finally ruled out.

When we performed this experiment with our evolutive approach, it created a race in front of each door and kept the races separated from each other all the time. This way, both races are really kept until new information is provided.

There are other solutions to solve this bias in the MCL, such as using omnidirectional cameras. We have preferred to improve the localization algorithm and still use regular monocular cameras as they appear more frequently in robot equipment, and other perceptive algorithms work with and require these regular cameras.

#### 7.2.2. Typical Execution in Humanoid Robot

The evolutionary algorithm has several parameters to be configured, such as the maximum number of races (10), the number of explorers (30), and the percentages of the genetic operators previously explained (20% elitism, 20% crossover and 60% mutation). The maximum number of races has been a crucial factor. A high number of races improves the accuracy of the calculated localization, but it also increases the algorithm execution time. We have selected a value, 10, that offers a good balance between efficiency and accuracy.

**Figure 21.**
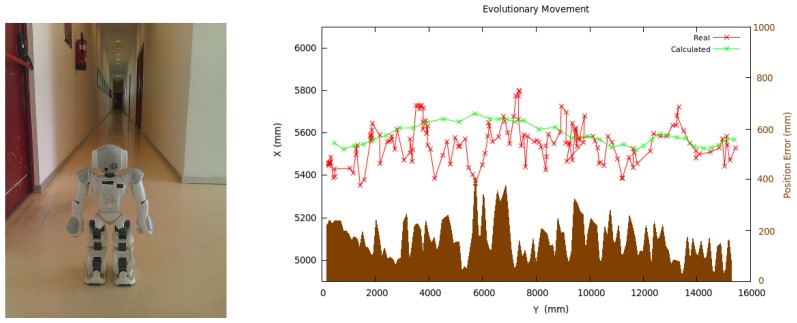
Nao robot traveling a corridor for experiments (left) and estimated localization and position error over time (right).

The first experiment was performed with a real Nao robot travelling through a corridor (Figure 21). It shows how the algorithm is able to follow the real movement of the robot starting on a known position. At first, the robot is located in a known position; afterwards, we move the robot around the environment and measure its localization error. The red line in Figure 21 shows the calculated positions, the green line the real robot path, and the brown area is the measured error. The average error was 11.8 cm and 2.1 degrees. The algorithm is able to follow the robot movement even when its instantaneous observations do not provide enough information due to robot odometry. Besides, we can emphasize that the trajectory followed by the robot is very stable and is always close to the real location of the robot. The ground truth location of the robot was calculated manually measuring the distance from the robot to known objects, such as doors or corridor lines. These distances were calculated each 5 seconds and interpolated in between.

The evolutionary visual localization algorithm runs in iterations. The average iteration time including all processing (health computation, race management, explorer creations, *etc.*) was 110 ms.

#### 7.2.3. Dealing with Symmetries and Kidnappings

**Figure 22.**
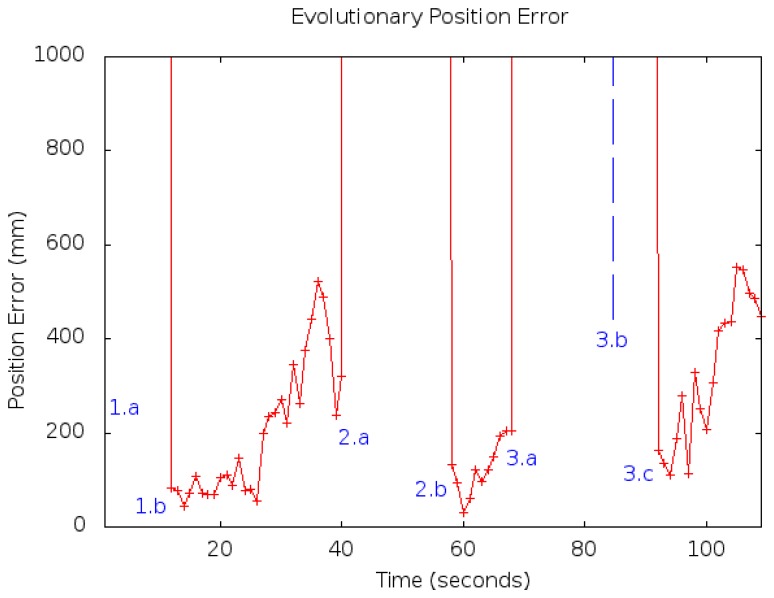
Position error over time.

The second experiment (Figure 22) shows how the algorithm works with symmetries and kidnappings. At first instant (1.a), we locate the robot in front of a door, so the algorithm creates several races where the robot may be located. The algorithm selects one of them (a wrong one) but keeps another one on the right location. Then the robot moves and obtains more information from the world and finally rules the wrong location out and selects the correct one (at 1.b instant). Afterwards, we kidnap the robot to another location (2.a) and it takes a while until the robot changes its estimation to the new right location. This happens because the location's reliability changes gradually to avoid changing with false positives, but after a while, it changes to the new position (2.b). A second kidnapping is performed (3.a), and this case is similar to the first one: first, it selects a wrong localization (3.b), but after some iterations, it changes to the correct one (3.c). The average error after selecting the correct race was 22.5 cm and 5.8 degrees. We also measured the time spent until the algorithm calculates a new plausible pose after a kidnapping (recovery time). This experiment took 21 secs.

#### 7.2.4. Health Function Based on Instantaneous Images

**Figure 23.**
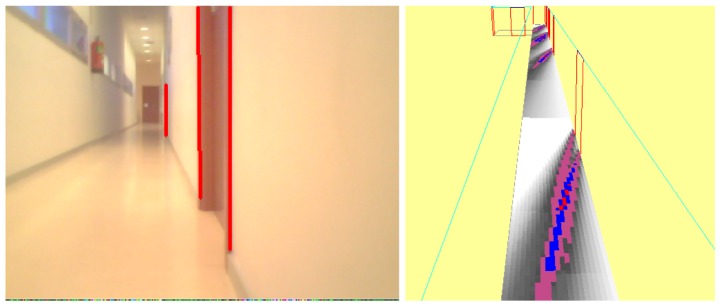
Observed image (left) and probabilities calculated with *theta* equals to 0 radians(right).

To validate the health function, we have implemented a debugging mechanism to show graphically the value returned by the health function in different positions. In Figure 23, we show the value returned in all positions (X, Y), where red areas are the ones with highest probability and white areas with the lowest. As we can see, the position with highest probability is likely with the input image.

**Figure 24.**
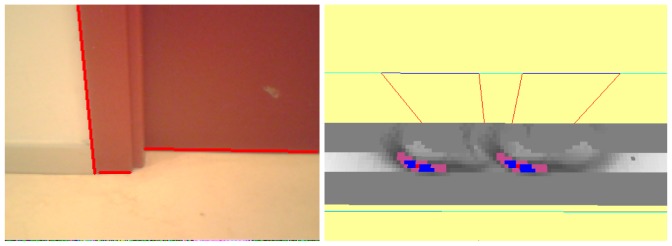
Observed image in front of a door (left) and probabilities calculated for any *theta* (right).

**Figure 25.**
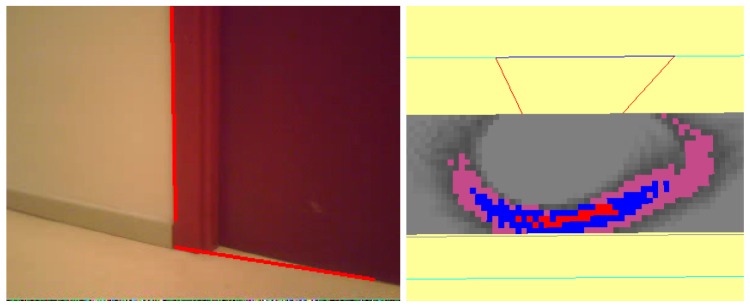
Observed image (left) and calculated probabilities without occlusions or false positives for any *theta* (right).

**Figure 26.**
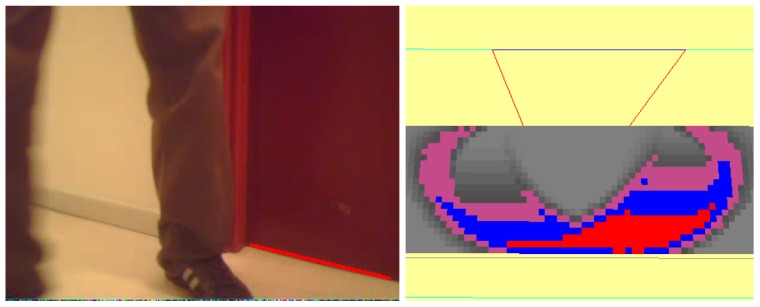
Observed image (left) and calculated probabilities with occlusions for any *theta* (right).

**Figure 27.**
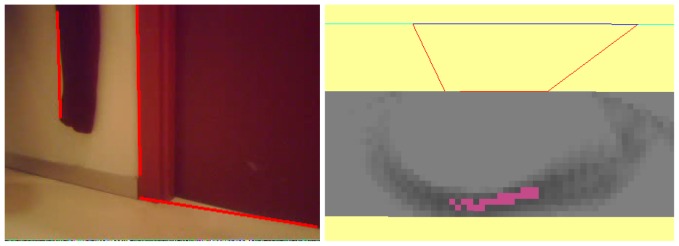
Observed image (left) and calculated probabilities with false positives for any *theta* (right).

In case of symmetries, we obtain high probabilities in several positions. In Figure 24 we show what would happen if we obtained an image in front of a door, then we would obtain high probabilities in front of each door of our environment:

The localization algorithm has been tested in more complex scenarios, with occlusions and false positives. In case of occlusions, the algorithm keeps a good behavior, since our approach does not penalize the probability if an object is not detected in the image when it should be. The light negative impact of occlusions is a higher probability in some more field areas (compare Figures 25 and 26). However, false positives affect very negatively health function and the calculated position can be totally wrong (compare Figures 25 and 27).

#### 7.2.5. Health Function Based on Visual Memory

In case of using visual memory instead of instantaneous images for the health function, the calculated values will be similar to previous health function, but we will get two benefits: there will be less symmetries, because we will get more information about the environment, and temporal occlusions will affect even less our health function.

We have performed an experiment to compare the localization algorithm with and without visual memory. We placed the robot in front of two consecutive doors but without observing both at the same time. At first, the robot only detects the first door, and afterwards, we move the robot sideways so that it no longer detects the first door and it starts observing the second one.

The localization algorithm without visual memory is not able to locate the robot always in the proper place. It creates a race in front of several doors once the first door is detected, but not in front of all of them. When the second door is observed, there may be two possibilities:

- If a race was created in front of two consecutive doors when the first door was observed, the algorithm would choose this race because the second door would fit with that position.
- If that was not the case, the algorithm would select a race in front of any door. Since it cannot remember that there was another door before, then, in most cases, it would pick up a wrong position.

**Figure 28.**
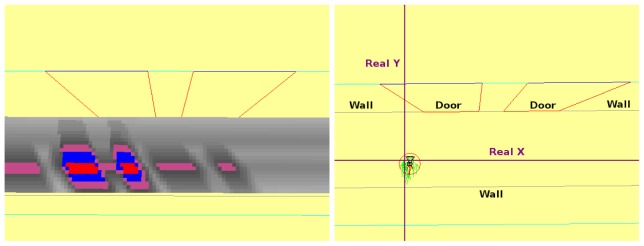
Health values for any *theta* (left) and estimated position (right) calculated with visual memory.

However, when the second door is detected using the visual memory, the algorithm would be able to select the correct race even in the second case, since it would not forget the first door. We show in Figure 28 the health map and position estimation calculated with the visual memory.

## 8. Conclusions

In this paper, a visual perception system for autonomous robots has been presented. It processes the images from a mobile camera and builds a short-term local memory with information about the objects around the robot, even if they lie outside the current field of view of the camera. This visual memory stores 3D segments and simple objects like parallelograms with their associated properties like position, uncertainty (inverse of *life*), color, *etc.* It allows better navigation decisions and even better localization as it includes more information than the current image, which can even be temporary occluded.

An overt visual attention mechanism has been created to continuously select where the mobile camera should look at. Using a *salience* dynamics and choosing the most salient point, the system shares the gaze control between the need to reobserve objects on the visual memory and the need to explore new areas, providing also inhibition of return.

We developed a visual self-localization technique that uses an evolutionary algorithm. It keeps a population of particles to represent tentative robot positions and the particle set evolves as new visual information is gathered or with robot movements. It has been especially designed to deal with symmetries, grouping particles into races. There is one race for each likely position and, inside it, individuals do the fine-grain search. It can work both with just the current image or the contents of the visual memory.

This visual perception system has been validated both on real robots and in simulation. The memory nicely represents the robot surroundings using the images from the mobile camera, which movement is controlled by our attention mechanism. The memory is dynamic but has some persistence to deal with temporary occlusions. The localization works in real-time, provides position errors below 15cm and 5 degrees and is robust enough to recover from kidnappings or estimation errors in symmetric environments.

We are working on extending the visual memory to manage stereo pairs and RGB-D sensors as inputs and to deal with objects at any 3D position, not just the floor. The localization algorithm is designed and ready for that, but the current visual memory implementation works with one regular camera and does not provide full 3D segments at any position—it assumes all the objects lie on the floor. This limitation also causes that painted lines or color changes on the floor, which are traversable, are wrongly considered as obstacles to avoid. More experiments are also required in more challenging scenarios, with more dynamic obstacles or people around the robot and with faster movements. We are also studying how to help the robot deal with more abstract objects like tables and chairs in the visual memory. Regarding localization, we are working on introducing a monoSLAM EKF for each race of the evolutionary algorithm and on improving it to extract localization information from abstract objects, not only 3D points.
